# Enhancement of Casimir Friction between Graphene-Covered Topological Insulator

**DOI:** 10.3390/nano12071148

**Published:** 2022-03-30

**Authors:** Ting Yu, Rong Luo, Tongbiao Wang, Dejian Zhang, Wenxing Liu, Tianbao Yu, Qinghua Liao

**Affiliations:** Department of Physics, Nanchang University, Nanchang 330031, China; Yutingncu157@163.com (T.Y.); luorong_jjvc@163.com (R.L.); zhangdejian2015@163.com (D.Z.); liuwenxing@ncu.edu.cn (W.L.); yutianbao@ncu.edu.cn (T.Y.); lqhua@ncu.edu.cn (Q.L.)

**Keywords:** casimir friction, graphene, topological insulator

## Abstract

Casimir friction is theoretically studied between graphene-covered undoped bismuth selenide (Bi_2_Se_3_) in detail. In the graphene/Bi_2_Se_3_ composite structure, the coupling of the hyperbolic phonon polaritons supported by Bi_2_Se_3_ with the surface plasmons supported by graphene can lead to the hybrid surface plasmon–phonon polaritons (SPPPs). Compared with that between undoped Bi_2_Se_3_, Casimir friction can be enhanced by more than one order of magnitude due to the contribution of SPPPs. It is found that the chemical potential that can be used to modulate the optical characteristic of SPPPs plays an important role in Casimir friction. In addition, the Casimir friction between doped Bi_2_Se_3_ is also studied. The friction coefficient between doped Bi_2_Se_3_ can even be larger than that between graphene-covered undoped Bi_2_Se_3_ for suitable chemical potential due to the contribution of unusual electron surface states. The results obtained in this work are not only beneficial to the study of Casimir frictions but also extend the research ranges of topological insulators.

## 1. Introduction

In nature, friction is a very common phenomenon, which extensively exists in the macroscopic to the microscopic world. However, the physical mechanism of friction in the microscopic world is different from that of the friction observed in our daily lives. In recent years, people have paid much attention to nanotribology with the rapid development of nanofabrication technology. Interestingly, there is one type of friction that appears between bodies in relative motion even without direct contact at the nanoscale, which is usually called noncontact friction [1,2,3,4,5,6,7]. Noncontact friction has been observed experimentally by atomic force microscopy [1,5,6] and has important practical significance in ultra-sensitive force detection. In the past decade, another noncontact friction originating from the momentum exchange of Doppler-shifted photons has attracted much attention [8]. The essence of this friction is closely related to the Casimir effect, so it is called Casimir friction [8,9]. Even at zero temperature, Casimir friction can still exist because of the vacuum fluctuation, so it will be denoted as quantum friction. Casimir frictions have been extensively studied in several configurations that are in relative motion, such as atom and atom [10], atom and plate [11,12,13], and plate and plate [14].

The theoretical method of Casimir friction between two objects separated by a small gap was firstly derived by Pendry with the help of classical electromagnetic theory [8]. Then, Volokitin and Persson also made a series of works on Casimir friction [15,16,17,18,19,20]. Because the Casimir friction is extremely small, it makes a great challenge to detect in an experiment. Enhancing the Casimir friction between bodies in relative motion becomes an important topic. An enhancement mechanism of Casimir friction associated with resonant photon tunneling on different surfaces was proposed [16]. It has been found that Casimir friction will be significantly enhanced if the materials can support low-frequency surface plasmons (SPs) or other surface polaritons [16]. Therefore, plenty of materials that can support surface polaritons are employed to investigate the Casimir friction [21,22]. Among them, graphene is a two-dimensional material composed of carbon atoms [23,24,25], which can support SPs from terahertz (THz) to infrared frequency ranges [26,27,28,29,30,31,32,33,34,35]. In particular, the optical conductivity of graphene depends on its chemical potential, which is controlled by an external field or gate voltage. It is shown that SPs supported by graphene can play an important role in enhancing and actively modulating Casimir friction [36,37,38,39]. In addition, the Casimir friction between graphene-covered hyperbolic materials (HMs) has also been studied [40]. The coupling of graphene plasmon with hyperbolic phonon polaritons (HPPs) supported by HMs can enhance the Casimir friction remarkably [40]. 

In the past decade, topological insulators have received extensive attention because of their exotic characteristic and important applications in the fabrications of new electronic devices [41,42,43,44,45]. They are characterized by a full insulating gap in the bulk and gapless surface states protected by time-reversal symmetry [44]. The three-dimensional (3D) topological insulators have been predicted theoretically in the Bi_1−x_Sb_x_ alloy [46] and observed experimentally by angle-resolved photoemission spectroscopy [47]. Then, several simple 3D topological insulators were predicted theoretically in Bi_2_Te_3_, Sb_2_Te_3_ [48], and Bi_2_Se_3_ [48,49] compounds with a large gap in bulk and gapless surface state. Bismuth-based topological insulators have attracted great interest due to their unusual electronic surface states, which are manifested as massless Dirac fermions [43,44,48,49,50,51]. As one of the representative topological insulators, Bi_2_Se_3_ is also well-known for its bulk optical response [52,53,54,55] besides its novel surface state. It exhibits hyperbolic dispersion relation and can support highly oriented collective HPPs in the THz range [56]. For Bi_2_Se_3_ with finite doping, the coupling of HPPs with Dirac plasmon confined at the surface can result in the appearance of hybrid modes [56]. However, the SPs are absent for undoped Bi_2_Se_3_. As has been mentioned above, graphene plasmon can be coupled with different surface polaritons, so it is interesting to investigate the coupling between graphene plasmon and HPPs supported by undoped Bi_2_Se_3_.

In this paper, we study the Casimir friction between graphene-covered undoped Bi_2_Se_3_. The low-frequency HPPs supported by Bi_2_Se_3_ can be coupled with SPs supported by graphene, and then resulting in the appearance of hybrid surface plasmon–phonon polaritons (SPPPs). We demonstrate that the SPPPs play an important role in the Casimir friction between graphene-covered Bi_2_Se_3_. Compared with that between undoped Bi_2_Se_3_, the Casimir friction between graphene-covered Bi_2_Se_3_ enhances about one order of magnitude in wide separation gaps. Furthermore, the hybrid SPPPs can be flexibly controlled by tuning the chemical potential of graphene that depends on the gate voltage. Therefore, it provides an opportunity to actively modulate the Casimir friction between topological insulators. Casimir friction between doped Bi_2_Se_3_ is also studied. Depending on the chemical potential, Casimir friction between doped Bi_2_Se_3_ can be larger or smaller than that between graphene-covered Bi_2_Se_3_ under the same separation distance. This study not only extends the research ranges of topological insulators but also provides an efficient method to control the Casimir friction.

## 2. Theoretical Model

**Figure 1:**
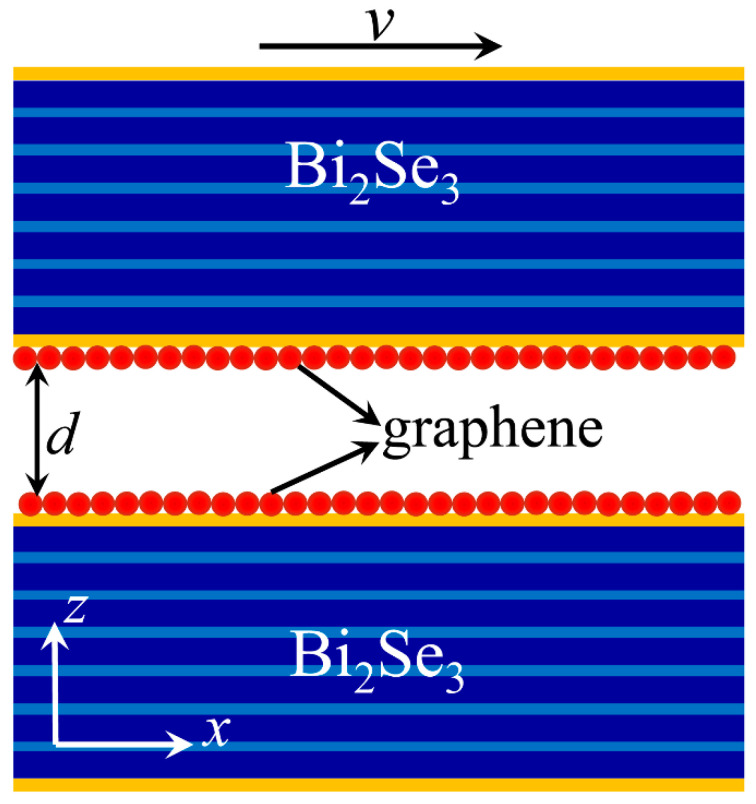
Schematic of Casimir friction between graphene-covered undoped Bi_2_Se_3_ separated by a distance *d*. The layered structure denotes Bi_2_Se_3_ and the two thin orange layers at the top and bottom of Bi_2_Se_3_ represent the surface states.

The schematic of the Casimir force between graphene-covered Bi_2_Se_3_ is shown in Figure 1. Two graphene-covered Bi_2_Se_3_ bulks are separated by a distance d. We assume the top graphene-covered Bi_2_Se_3_ moves relatively to the bottom one along the *x*-axis with a velocity v in the laboratory frame. The temperature of the surrounding environment is denoted by *T*, which is set to be *T* = 300 K in all the calculations. If the moving velocity of the top graphene-covered Bi_2_Se_3_ satisfies the conditions $v<dk_{B}T/\hbar$, Casimir friction is proportional to the sliding velocity, which can be determined as f=γv [18]. $k_{B}$ and ℏ are the Boltzmann and reduced Planck constants, respectively. $\gamma =\gamma^{\mathrm{rad}}+\gamma^{\mathrm{evan}}$ is the Casimir friction coefficient, which comes from the contributions of propagation and evanescent electromagnetic waves from the graphene-covered Bi_2_Se_3_. However, the contribution of the propagation wave can be ignored in the near-field region [2,18]. Therefore, we only consider the Casimir friction coefficient from the evanescent waves, which can be expressed as [18]

(1) γ≈ℏ2π2∫0∞dω−∂n∂ω∫ω/c∞dqq3e−2kzdImR1pImR2p1−e−2kzdR1pR2p2+p→s

where $n\left(\omega \right)=1/\left(e^{\hbar \omega /k_{B}T}-1\right)$ is the Bose–Einstein factor. q and $k_{z}=\sqrt{{\left(\omega /c\right)}^{2}-q^{2}}$ are the components of the wave vector components parallel and perpendicular to the *xy* plane, respectively. c is the speed of light in the vacuum and $R_{\mathrm{ip}}$ (i = 1,2) is the reflection amplitude from the top or bottom surface of the *p*-polarized electromagnetic waves. The symbol $\left[p\to s\right]$ represents the *p*-polarized reflection amplitude $R_{p}$ replaced with *s*-polarized reflection amplitude $R_{s}$. As described in Ref. [57], *p*-polarized wave often plays a dominant role in the near-field region, so the contribution from *s*-polarized waves can be excluded. The integral of *q* in Equation (1) is defined as force spectral density (FSD), which can be expressed by

(2) fp=−∂n∂ω∫ω/c∞dqq3ImR1pImR2p1−e−2kzdR1pR2p2e−2kzd

For convenient description, we define the photon exchange function as

(3) ξp=ImR1pImR2p1−e−2kzdR1pR2p2e−2kzd

which is used to describe the exchange ability of photons between the bodies in relative motion. The reflection amplitude of graphene-covered anisotropic material can be written as [58]

(4) $$\;R_{p}=\frac{p\varepsilon^{⊥}-p_{p}+\mu_{0}c\sigma pp_{p}}{p\varepsilon^{⊥}+p_{p}+\mu_{0}c\sigma pp_{p}}$$

where $p=\sqrt{1-\kappa^{2}}$, $p_{p}=\sqrt{\varepsilon^{⊥}-\varepsilon^{⊥}\kappa^{2}/\varepsilon^{z}}$, and κ=cq/ω. $\varepsilon^{z}$ and $\;\varepsilon^{⊥}$ are the permittivities of Bi_2_Se_3_ parallel and perpendicular to the optical axis (*z*-axis), respectively. $\mu_{0}\;$ is the permeability in the vacuum. $\sigma \left(\omega \right)$ is the optical conductivity of graphene, which can be simplified to $\sigma =\frac{ie^{2}\mu }{\pi \hbar^{2}\left(\omega +i\tau^{-1}\right)}\;$ in the low-frequency range by ignoring the contribution of interband transitions [32]. *e* is the electron charge, μ  is the chemical potential of graphene, and $\tau =10^{-13}$ s is the relaxation time. As a naturally anisotropic material, Bi_2_Se_3_ exhibits hyperbolic optical properties. The dielectric properties perpendicular ($\varepsilon^{⊥}$) and parallel ($\varepsilon^{z}$) to the optical axis have different values which are given by [59,60]

(5) $$\varepsilon^{\alpha }\left(\omega \right)=\varepsilon_{\infty }^{\alpha }+\underset{j=1,2}{\sum }\frac{\omega_{p,j}^{\alpha \;2}}{\omega_{\mathrm{to},j}^{\alpha \;2}-\omega^{2}-i\gamma_{j}^{\alpha }\omega },\left(\alpha =⊥,z\right)$$

**Figure 2:**
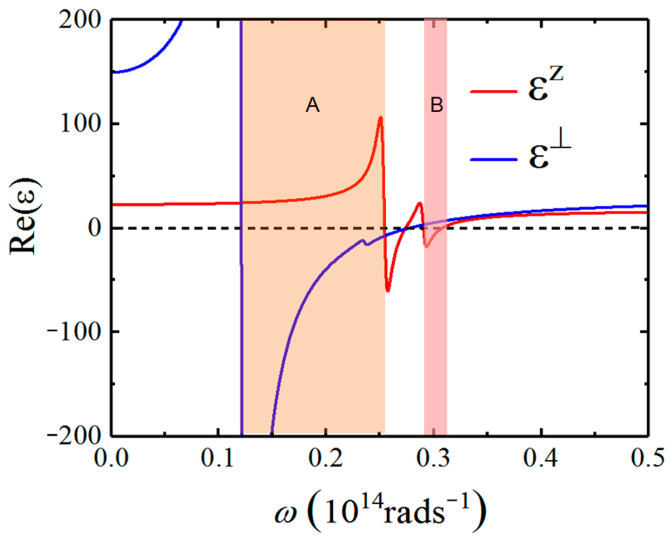
The real part of the dielectric function of Bi_2_Se_3_. The shaded regions denote the hyperbolic regions. Regions A and B denote type II (Reε⊥0, Reεz0) and type I (Reε⊥>0, Reεz<0 ) hyperbolic bands, respectively.

The parameters used in Equation (5) are $\varepsilon_{\infty }^{⊥}=29$, $\varepsilon_{\infty }^{z}=17.4$, ${\;\omega }_{\mathrm{to},1}^{⊥}=64{\;\mathrm{cm}}^{-1\;}$, $\omega_{p,1}^{⊥}=704{\;\mathrm{cm}}^{-1}$, $\omega_{\mathrm{to},2}^{⊥}=125{\;\mathrm{cm}}^{-1}$, ${\;\omega }_{p,2}^{⊥}=55{\;\mathrm{cm}}^{-1}$, $\;\omega_{\mathrm{to},1}^{z}=135{\;\mathrm{cm}}^{-1}$, $\omega_{p,1}^{z}=283\;{\mathrm{cm}}^{-1}\;$, $\omega_{\mathrm{to},2}^{z}=154{\;\mathrm{cm}}^{-1}$, $\omega_{p,2}^{z}=156{\;\mathrm{cm}}^{-1}\;$, $\gamma_{j}^{\alpha }=3.5{\;\mathrm{cm}}^{-1}$. The real parts of the dielectric functions $\varepsilon^{⊥}\left(\omega \right)$ and $\varepsilon^{z}\left(\omega \right)$ of Bi_2_Se_3_ are shown in Figure 2. There are two shaded regions, including regions A ($\omega_{\mathrm{to},1}^{⊥}<\omega <\omega_{\mathrm{to},1}^{z}$) where Bi_2_Se_3_ is type II HM (Re($\varepsilon^{z}$) > 0, Re($\varepsilon^{⊥}$) < 0) and region B ($\omega_{\mathrm{to},2}^{z}<\omega <163\;{\mathrm{cm}}^{-1}$) where Bi_2_Se_3_ is type I HM (Re($\varepsilon^{z}$) < 0, Re($\varepsilon^{⊥}$) > 0). Therefore, HPPs can be excited in these hyperbolic regions [56]. Although as a typical topological insulator, Bi_2_Se_3_ possesses unusual electron surface states, we firstly only consider its bulk optical response. When the frequencies are smaller than the bulk gap of 0.3 eV of Bi_2_Se_3_, the electronic contribution to permittivities (appearing in Equation (5) via $\varepsilon_{\infty }^{z}$) is purely real. In addition, it is assumed that the valence bulk band is completely filled and the conduction band is empty, there are no free carriers present in the bulk. The doping electron surface states of Bi_2_Se_3_ is determined by its chemical potential $\mu_{B}$ that is located inside the bulk band gap. The electron surface states can be excluded by setting $\mu_{B}=0.0$ eV.

## 3. Casimir Friction between Graphene-Covered Bi_2_Se_3_

**Figure 3:**
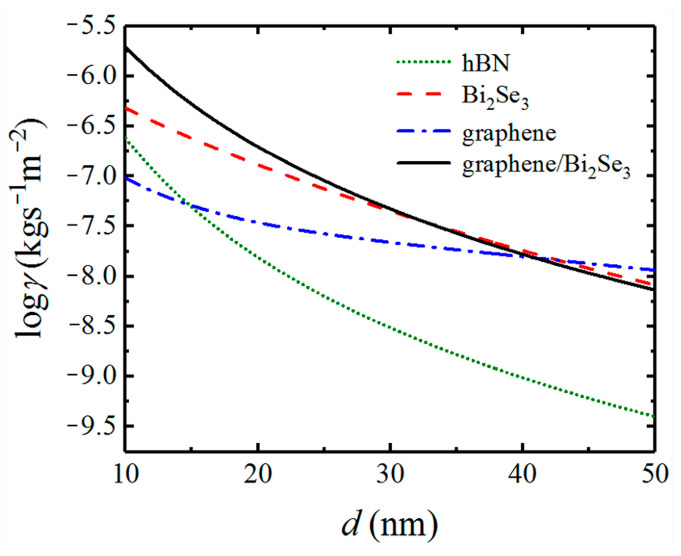
The dependence of Casimir friction coefficients for different configurations on separation distance *d*. The chemical potential of graphene is μ=0.2 eV. The base of the logarithm is 10 in this and the following figures.

In Figure 3, we show the dependences of Casimir friction on distance d for different configurations. As a natural hyperbolic material, hBN can also support HPPs which are located in the infrared regions. The permittivities of hBN can be found in Ref. [58]. Here we also show the Casimir friction between hBN bulk for comparison. The results of hBN, Bi_2_Se_3_, graphene, and graphene-covered Bi_2_Se_3_ are denoted in green dotted, red dashed, blue dot-dashed, and black solid lines, respectively. The chemical potential of graphene is fixed at μ=0.2 eV. It is seen that the Casimir friction coefficients of different configurations decrease as the distance increases. The friction coefficients of graphene and Bi_2_Se_3_ decrease slowly as the distance increases, while the friction coefficients of hBN and graphene-covered Bi_2_Se_3_ decrease rapidly as the distance increases. Although the friction coefficient of Bi_2_Se_3_ is smaller than that of hBN when the distance is less than 25 nm, it has an obvious enhancement when covered with graphene. Compared with that of Bi_2_Se_3_, the friction coefficient between graphene-covered Bi_2_Se_3_ can increase more than one order of magnitude when the chemical potential of graphene is 0.2 eV.

**Figure 4:**
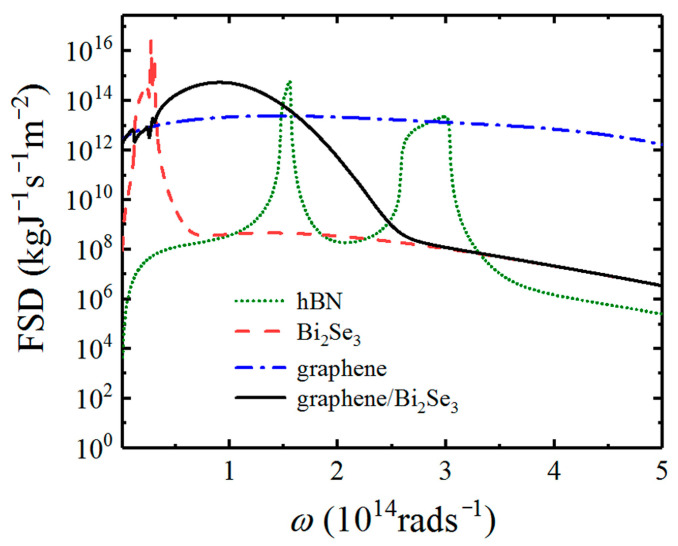
The force spectral densities for different configurations. The parameters used here are the same as those used in Figure 3.

Because Casimir friction is mainly from the exchange of the evanescent waves in the near-field region, we display the FSDs for different configurations to realize the contribution of different evanescent waves to Casimir friction in Figure 4. The results of hBN, Bi_2_Se_3_, graphene, and graphene-covered Bi_2_Se_3_ are displayed in green dotted, red dashed, blue dot-dashed, and black solid lines, respectively. The chemical potential of graphene is set to be μ=0.2 eV and the separation distance is d=20  nm. It can be clearly seen from Figure 4 that there are two peaks corresponding to its hyperbolic bands located in the infrared frequency region for the FSD of hBN. Similarly, there are two peaks in the low-frequency regions for Bi_2_Se_3_. FSD has a strong peak corresponding to the resonance frequency of the HPPs on the surface of Bi_2_Se_3_ material, so the main contribution to quantum friction in Bi_2_Se_3_ comes from the HPPs. The FSD spectrum of graphene covers a very wide frequency range but with a relatively low value, which agrees that graphene can support *p*-polarized SPs with a wide frequency range. For graphene-covered undoped Bi_2_Se_3_, the HPPs supported by Bi_2_Se_3_ can be coupled with SPs supported by graphene, which results in the shift of the hybrid SPPPs toward higher frequencies. Therefore, the Casimir friction between graphene-covered Bi_2_Se_3_ is mainly from the SPPPs. We can qualitatively obtain the Casimir friction by judging the area covered by the curves of different configurations. 

**Figure 5:**
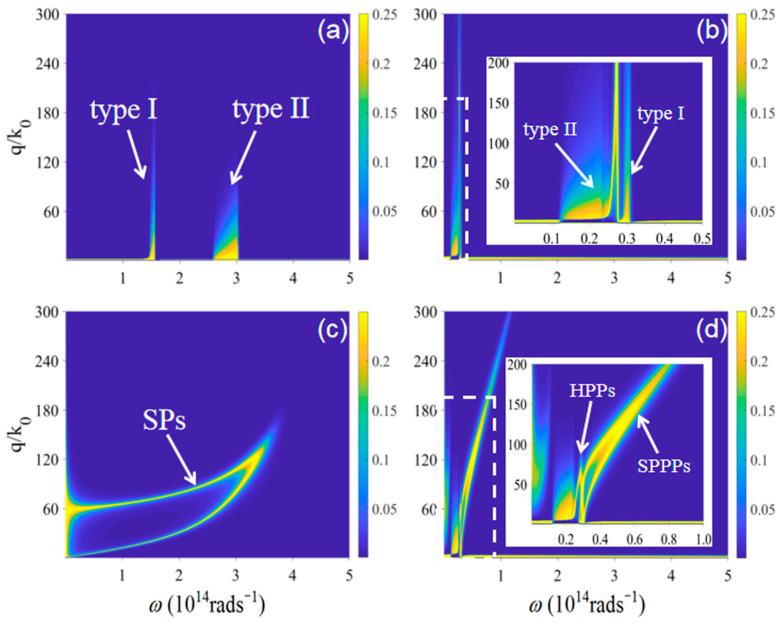
The photon exchange functions in different configurations. (**a**): hBN; (**b**): Bi_2_Se_3_; (**c**): graphene; (**d**): graphene-covered Bi_2_Se_3_. The chemical potential of graphene is μ=0.2 eV and the separation distance is d=20 nm. The insets in (**b**,**d**) are the enlarged areas denoted by white dashed lines.

To further analyze the physical mechanism of Casimir frictions for different configurations, we show the photon exchange functions of these configurations in Figure 5. The bright regions in the dark background denote the different surface polaritons supported by different materials. In Figure 5a, the bright regions are from the contributions of type I and type II hyperbolic bands of hBN, respectively. For the case of Bi_2_Se_3_ shown in Figure 5b. we can see that the hyperbolic bands also exist in the THz frequency range. For the photon exchange function between graphene sheets shown in Figure 5c, it covers a very wide frequency range since graphene can support SPs from THz to infrared frequencies. When Bi_2_Se_3_ is covered with graphene, the HPPs supported by Bi_2_Se_3_ can couple with SPs supported by graphene to form the SPPPs which is out of the hyperbolic band. The SPPPs move toward higher frequencies as the wave vector increases. Therefore, we can obtain that SPPPs dominate the Casimir friction between graphene-covered undoped Bi_2_Se_3_.

**Figure 6:**
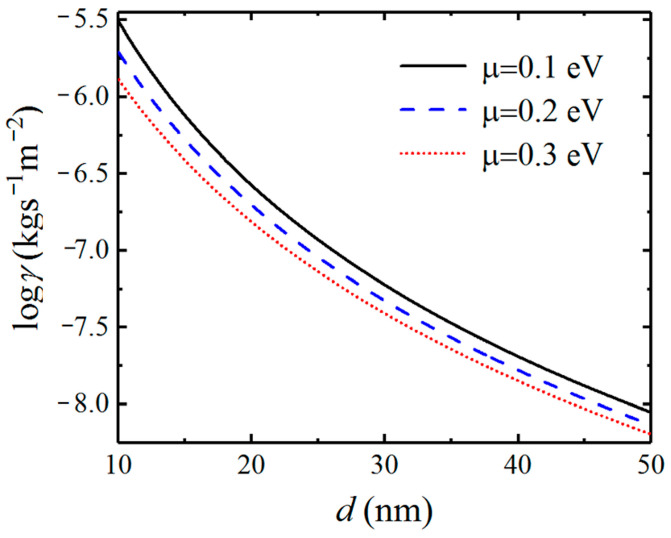
Dependence of the Casimir friction coefficient between graphene-covered Bi_2_Se_3_ on the separation distance for different chemical potentials of graphene.

Because the characteristic of SPs excited by graphene as well as the coupled SPPPs is dependent on the chemical potential of graphene, we investigate the effect of chemical potential on the Casimir friction. In Figure 6, we display the friction coefficient on the separation distance d for different chemical potentials of graphene. The friction coefficient between graphene-covered Bi_2_Se_3_ with chemical potentials μ=0.1, μ=0.2, and μ=0.3 eV are plotted in black solid, blue dashed, and red dotted lines, respectively. We can see that the friction coefficients varying with distance exhibit different behavior for different chemical potentials. As the distance increases, the difference of Casimir friction between systems with different chemical potentials becomes smaller, which means that the contribution from the evanescent SPPPs becomes weaker as the distance increases.

**Figure 7:**
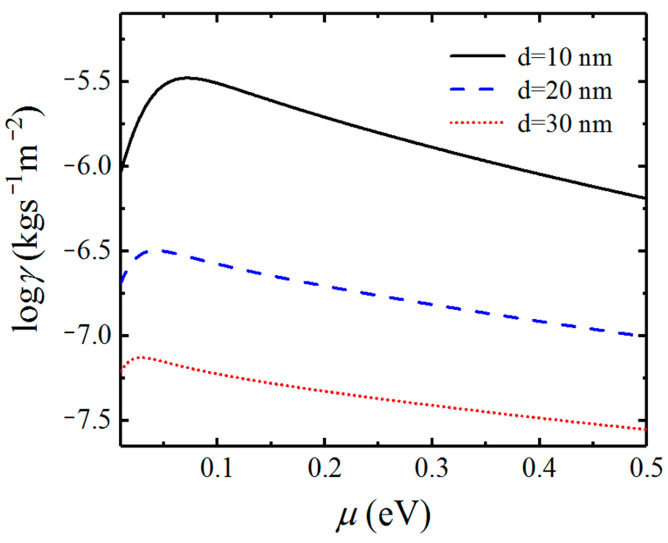
Dependence of the Casimir friction coefficient on chemical potential for different separation distances d.

In Figure 7, we show the dependence of the friction coefficient on the chemical potential between graphene-covered Bi_2_Se_3_ bulks. The friction coefficients with separation distances of 10, 20, and 30 nm are plotted in black solid, blue dashed, and red dotted lines, respectively. In Figure 7, we can see that the friction coefficients increase first and then decrease after reaching the maximum values as the chemical potential increases. In particular, the difference between the maximum value and minimum value of friction coefficients can reach about one order of magnitude for different chemical potentials for the separation distance being 10 nm. When the separation distance increases, the maximum and minimum values of Casimir friction still have an obvious difference in our considered chemical potential range. Therefore, the maximum value of the friction coefficient can be obtained by tuning the chemical potential of graphene.

**Figure 8:**
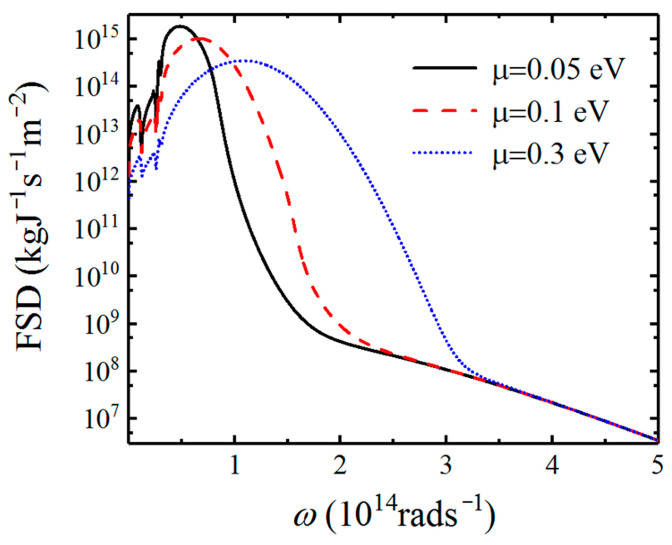
Force spectral densities of graphene-covered Bi_2_Se_3_ as a function of angular frequency for different chemical potentials at a fixed distance d=20 nm.

In Figure 8, we show the FSD in order to understand the physical mechanism of the relationship between the friction coefficient and chemical potential. The separation distance between the top and bottom graphene-covered Bi_2_Se_3_ is fixed at d=20 nm in the calculations. When the chemical potential of graphene is smaller (μ=0.05 eV), we can see that Casimir friction is mainly determined by the HPPs as shown by the solid black line. In this case, the coupling between HPPs and graphene plasmons is weak. The HPPs and graphene plasmon contribute to the Casimir friction separately. As the chemical potential increases (μ=0.1 eV), the coupling between HPPs and SPs occurs, resulting in the enhancement of SPPPs in a wide frequency region compared with the SPs supported by graphene as shown in red dashed lines in Figure 8. However, when the chemical potential continues to increase, the peak of SPPPs shifts toward higher frequencies with a decrease in magnitude. This will lead to the decrease in the Casimir friction. Therefore, we can demonstrate the coupling of graphene plasmon with HPPs supported by undoped Bi_2_Se_3_ can enhance the Casimir friction significantly between graphene-covered Bi_2_Se_3_. It is also possible to actively modulate the Casimir friction between such graphene/Bi_2_Se_3_ composite structures by controlling the gate voltage.

## 4. Casimir Friction between Doped Bi_2_Se_3_ Bulks

We have investigated the Casimir friction between graphene-covered undoped Bi_2_Se_3_ in the previous sections. Then, we continue to study the Casimir friction between doped Bi_2_Se_3_ without covering graphene. Such a configuration can be realized by moving graphene away in Figure 1. In this configuration, the Bi_2_Se_3_ bulk behaves like an anisotropic material, while the two layers denoted in orange represent the top and bottom surface states. The electron surface states which behave as massless Dirac fermions exist when Bi_2_Se_3_ is doped; it plays a similar role as graphene in the graphene-covered undoped Bi_2_Se_3_ configuration. The fundamental density response functions of the surface states are the sheet conductivity $\sigma_{B}$ and polarizability P, which have the following relation [56]

(6) $$\sigma_{B}\left(q,\omega \right)=\frac{i\omega }{q^{2}}e^{2}P\left(q,\omega \right)$$

Under the condition of random-phase approximation for Dirac fermions, $P\left(q,\omega \right)$ can be obtained analytically [56]:

(7) $$P\left(q,\omega \right)=-\frac{Nk_{F}}{2\pi \hbar v_{F}}-\frac{iN}{16\pi \hbar v_{F}}\frac{q^{2}}{\sqrt{q^{2}-k_{\omega }^{2}}}[G(\frac{k_{\omega }+2k_{F}}{q})-G(\frac{k_{\omega }-2k_{F}}{q})-i\pi ]$$

where function $G\left(x\right)$ are expressed as $G\left(x\right)=ix\sqrt{1-x^{2}}-i\arccos x$. N=1 is the number of Dirac cones. $k_{F}=\left|\mu_{B}\right|/\hbar v_{F}$ is the Fermi momentum with $v_{F}=\sqrt{\mu_{\mathrm{dc}}\mu_{B}\tau_{B}/e}$ being the Fermi velocity, and $\mu_{\mathrm{dc}}$ is the electron mobility. $k_{\omega }=\left(\omega +i\gamma_{e}\right)/v_{F}$ and $\gamma_{e}>0$ is a phenomenological parameter that stands for electron scattering rate. After substituting the conductivity in Equation (4) with $\sigma_{B}$ in Equation (6), we can calculate the Casimir friction between doped Bi_2_Se_3_ by employing Equation (1).

**Figure 9:**
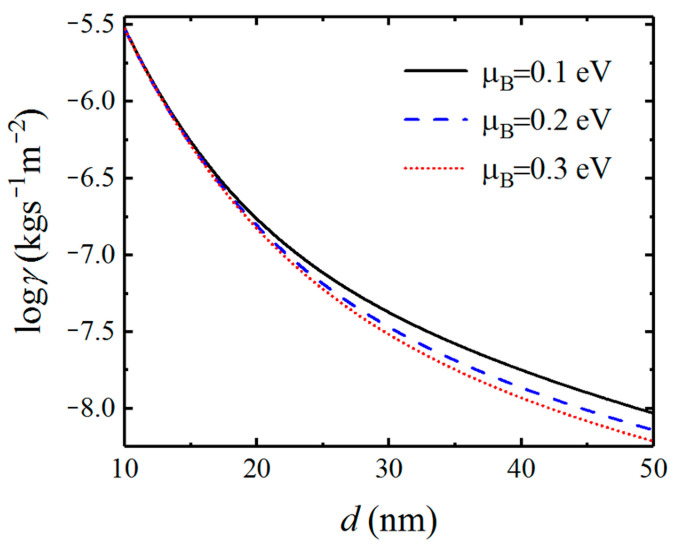
Dependence of the Casimir friction coefficient on the separation distances for different chemical potentials of Bi_2_Se_3_.

In Figure 9, we show the dependence of the Casimir friction coefficient on the separation distance d for different chemical potentials $\mu_{B}$ of doped Bi_2_Se_3_, which is similar to that shown in Figure 6. We can see that the Casimir friction coefficient decreases rapidly with the increase in separation distance d. When the separation distance is less than 20 nm, the friction coefficient is not sensitive to the chemical potential. When the separation distance becomes larger, the friction coefficient decreases as the chemical potential increases. Such phenomenon is different from that between graphene-covered undoped Bi_2_Se_3_ which is shown in Figure 6. In addition, comparing Figure 9 with Figure 3, we can also see that the friction coefficient between doped Bi_2_Se_3_ is larger than that between undoped Bi_2_Se_3_ when the separation distance is less than 20 nm. However, when the separation is larger than 20 nm, the difference in Casimir friction between undoped Bi_2_Se_3_ and doped Bi_2_Se_3_ is very small.

**Figure 10:**
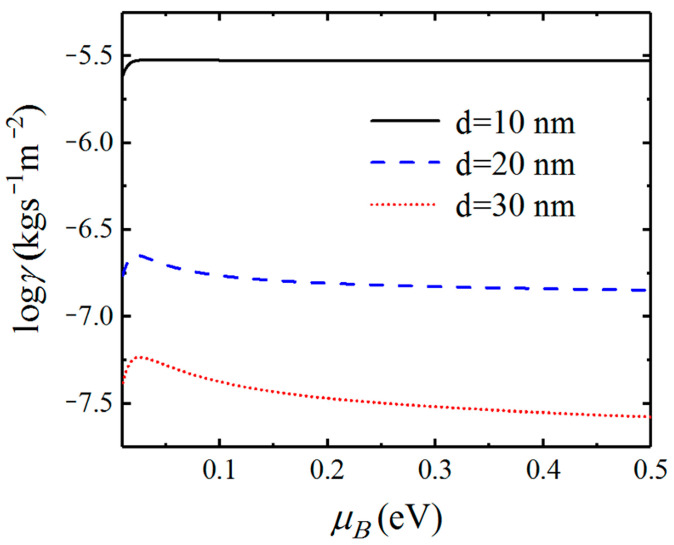
Dependence of the Casimir friction coefficient on chemical potential for different separation distances d.

In Figure 10, we show the dependence of the Casimir friction coefficient on the chemical potential of doped Bi_2_Se_3_ for different separation distances d. When the separation distance is 10 nm, the friction coefficient almost does not vary with the chemical potential. That means the chemical potential of doped Bi_2_Se_3_ has little impact on the surface states that dominate the Casimir friction at such a small distance. The Casimir friction, in this case, is even larger than that between graphene-covered Bi_2_Se_3_ when the chemical potential is larger than 0.1 eV, which can be seen in Figure 7. For the cases with separation distances are 20 and 30 nm, the friction coefficients first increase and then decrease after reaching the maximum values as the chemical potential $\mu_{B}$ increase. However, the friction coefficients between doped Bi_2_Se_3_ are not as sensitive to the chemical potential as those between graphene-covered undoped Bi_2_Se_3_.

## 5. Conclusions

In summary, we have studied the Casimir friction between graphene-covered Bi_2_Se_3_. Owing to the coupling of the HPPs supported by Bi_2_Se_3_ to the SPs excited by graphene, the Casimir friction between the proposed structures shows a significant enhancement compared to those between undoped Bi_2_Se_3_ materials. The Casimir friction can be modulated actively by tuning the chemical potential of graphene. It is found that the maximum value of Casimir friction can be obtained by choosing the appropriate chemical potential. Furthermore, Casimir friction between doped Bi_2_Se_3_ is also studied. When the chemical potential is larger than 0.1 eV, Casimir friction between doped Bi_2_Se_3_ is even larger than that between graphene-covered undoped Bi_2_Se_3_. Although more and more schemes have been proposed to enhance the Casimir force, it is still challenging to observe such a small force exactly in an experiment. Because it may play an important role in the nanoelectromechanical systems (NEMS), it is feasible to observe the Casimir force in the NEMS successfully. The results of this study are of great help to extend the research range of Casimir frictions and are meaningful to understanding the application of ultra-sensitive force detection of topological insulators.

## Data Availability

The data that support the findings of this study are available upon reasonable request from the corresponding author.
